# The Superior Royal School of Veterinary Medicine of Milan

**Published:** 1885-01

**Authors:** 


THE SUPERIOR ROYAL SCHOOL OF VETERINARY MEDICINE OF MILAN.—The report for the above institution for the year 1884 is quite an extended one of nearly 200 pages, and has some fine illustrations. It opens with a historical description of the school by Prof. Langellotti-Buonsanti, its Director. He tells how veterinary medicine was first taught as a science in France in the latter half of the Eighteenth Century by Claudio Bourgelat, who founded two schools in less than a decade, one in Lyons and one in Paris. The news soon spread throughout Europe of the immense benefits which agriculture derived from the establishment of these schools. Lombardy could not remain indifferent to the question, and the government sent two *talented persons* to Lyons to study the science of Veterinary Medicine, as in no other country was it more necessary than in this. The proposition ran thus: "Cultivators are men most precious to the State, and the cattle in which they are so rich, are indispensable for the sustaining of that most noble, useful and ancient art, Agriculture, and we cannot without pain and fear see those animals sick or wounded, exposed to die without assistance, or perhaps abandoned to the blind superstitious practices of persons without principles or education."

Having completed their studies these gentlemen returned to Milan, and the school was opened in 1791.

The course of instruction occupies four years and is distributed as follows:

First Year.—Botany, Zoölogy, Chemistry (organic and inorganic), Descriptive Anatomy, Experimental Physiology.

Second Year.—Descriptive Anatomy, Experimental Physiology, Exterior Configuration of the Animals, Race, Hygiene (first part).

Third Year.—General Pathology and Pathological Anatomy, Materia Medica, Hygiene (part second), Podology, Topographical Anatomy, Medical Pathology, Surgical Pathology, Operative Medicine, Clinical Medicine, Clinical Surgery.

Fourth Year.—Medical Pathology, Surgical Pathology, Clinical Medicine, Clinical Surgery, Operatory Medicine, Obstetrics, Commercial Veterinary Jurisprudence, Zoötechnics, Clinic for Cows and Swine.

The clinics are divided into three services: Stable clinic, Polyclinic, and Ambulatory clinic.

The first is intended to supply the students with practical materials for study, and to serve as a model hospital for the cure of sick animals where the results of treatment can be seen, and the use and mode of application of new instruments can be learned, especially for the purpose of stimulating research in the most important branch of veterinary science: viz., contagious diseases.

The second proposes to give the students a practical knowledge of the daily routine of cases, making diagnosis, prescribing, etc.

The ambulatory service is found only in this school. It is intended for the fourth year students, and consists of a series of excursions into the country in company with a professor. Sick animals are treated, operations performed, and lectures given in relation to all matters of interest to the student, reproduction, confinements, etc. Microscopical examinations are also made.

The Library contains 4500 volumes. The Laboratory is arranged so that each student can avail himself of all the facilities it affords for complete investigations in any branch of veterinary science.

The Anatomical Museum is complete. Among the objects of interest it contains the skeleton of an Arab horse used by Napoleon in the Egyptian campaign.

The Report closes with a long list of scientific publications by the professors. C.

